# Suicide Left Ventricle During Lung Transplantation in a Patient With Hereditary Hemochromatosis

**DOI:** 10.7759/cureus.116231

**Published:** 2026-09-14

**Authors:** Vitoria Simas, Timothy Waite, Edwin Becher, Nicholas Cavanaugh, Makoto Kataoka, Zachary Cohen, Satoshi Hanada, Megan Carmony, Chadrick Denlinger, Nuo Yang

**Affiliations:** 1 Department of Medicine, Indiana University School of Medicine, Indianapolis, USA; 2 Department of Anesthesia, Indiana University School of Medicine, Indianapolis, USA; 3 Department of Cardiothoracic Surgery, Indiana University School of Medicine, Indianapolis, USA

**Keywords:** acute heart failure, cardiogenic shock, hereditary hemochromatosis, left ventricular outflow tract obstruction, lung transplant, suicide left ventricle, systolic anterior motion of mitral valve leaflet, va-ecmo

## Abstract

Suicide left ventricle (SLV) is a rare cause of hemodynamic collapse due to dynamic left ventricular outflow tract (LVOT) obstruction, usually after surgical or transcatheter aortic valve replacement. We report a 54-year-old man with hereditary hemochromatosis undergoing bilateral lung transplantation. Following the weaning of venoarterial extracorporeal membrane oxygenation, he developed acute severe hypotension refractory to norepinephrine, vasopressin, and epinephrine. Intraoperative transesophageal echocardiography demonstrated a small left ventricular cavity, systolic anterior motion of the mitral valve, and LVOT obstruction, consistent with SLV. Subsequent treatment with very high-dose phenylephrine reversed and stabilized his hemodynamics, and the patient was ultimately discharged home with recovery of cardiac and pulmonary function. This case report presents an unusual occurrence of SLV in the absence of aortic valve intervention and highlights the key importance of clinical vigilance, early detection, and prompt appropriate treatment in this rare but life-threatening situation.

## Introduction

Suicide left ventricle (SLV) is a rare cause of hemodynamic collapse resulting from a left ventricular outflow tract (LVOT) obstruction. This complication is most commonly reported after transcatheter or surgical aortic valve replacement, where sudden relief of chronic afterload causes a hyperdynamic left ventricle [1,2], and vigorous ventricular contraction significantly reduces ventricular cavity size during systole. Moreover, high-velocity flow through the LVOT produces a Venturi effect that draws the anterior mitral leaflet toward the interventricular septum, producing systolic anterior motion (SAM) of the mitral valve [3]. The combination of both left ventricular cavity obliteration and SAM further exacerbates LVOT obstruction. Clinically, this vicious cycle of SLV presents as sudden severe hypotension despite preserved or hyperdynamic left ventricular function. Delayed treatment can lead to de novo right heart failure, cardiogenic shock, or even death [2]. Risk factors include female sex, a small left ventricle, significant septal or ventricular hypertrophy, and preexisting SAM of the mitral valve [4].

Here, we present an unexpected SLV event during bilateral lung transplantation in a patient with hemochromatosis when no valvular procedures were involved. Hereditary hemochromatosis (HH) is a common genetic metabolic disorder characterized by excessive iron absorption and elevated serum iron levels, which accumulate in and damage various organs, such as the heart, liver, and pancreas [4]. Heart failure and hepatic carcinoma are leading causes of mortality in HH, and approximately one-third of patients succumb to death as a result of cardiac causes [4,5]. Cardiac dysfunction occurs through myocardial iron deposition, which allows oxidative stress and subsequent cardiomyocyte injury, ultimately leading to iron-overload cardiomyopathy, including a hypertrophied septum. Epidemiologic studies demonstrate that patients with hemochromatosis have an increased risk of developing cardiomyopathy compared to the general population [6]. Cardiac manifestations initially present as restrictive cardiomyopathy with diastolic dysfunction due to increased ventricular stiffness that may then progress to dilated cardiomyopathy with systolic dysfunction [4].

This case demonstrates the critical importance of high vigilance for SLV, close monitoring with effective tools such as transesophageal echocardiography (TEE), and prompt targeted treatment, especially in lesser known clinical scenarios.

## Case presentation

A 54-year-old man with a weight of 75 kg and a height of 172 cm (body mass index of 25.4 kg/m^2^) presented for bilateral lung transplantation due to idiopathic pulmonary fibrosis. His past medical history was significant for HH and alpha-1 antitrypsin deficiency, status post liver transplantation nine years ago due to cirrhosis. He is currently on 1 mg of tacrolimus twice a day for immunosuppression, and his orthotopic liver has been functioning well with no rejection and normal hepatic function tests. His baseline oxygen requirement was 8 L at rest and 35 L with exercise. He had been hospitalized for three weeks for acute-on-chronic respiratory failure due to pulmonary embolism, which resolved with medical management. Preoperative pulmonary function test revealed a restrictive pattern. Cardiac catheterization 20 days prior to his lung transplantation showed normal coronary arteries, normal right and left heart pressures (mean RV pressure at 0 mmHg and mean pulmonary capillary wedge pressure at 1 mmHg), normal cardiac output (cardiac index at 3.9 L/min/m^2^), and normal pulmonary artery (PA) pressure (23/8 with mean at 14 mmHg). Transthoracic echocardiography (TTE) demonstrated preserved left ventricular systolic function (ejection fraction of 60%), grade I diastolic dysfunction, and normal right ventricular systolic function without significant valvular pathologies.

General anesthesia was induced, and the patient was successfully intubated with a single-lumen endotracheal tube due to a history of airway stenosis following prior bilateral thyroplasty. Lung isolation was achieved by sequential advancement of the endotracheal tube into the main bronchus to facilitate single-lung ventilation. Invasive monitoring was established, including radial and femoral arterial lines, central lines, a PA catheter, and TEE. Initial intraoperative TEE after induction revealed findings consistent with his preoperative TTE. In retrospect, there was a hypertrophic septum measuring 2.2 cm in maximum diameter that should have been of note (Figure 1).

**Figure 1:**
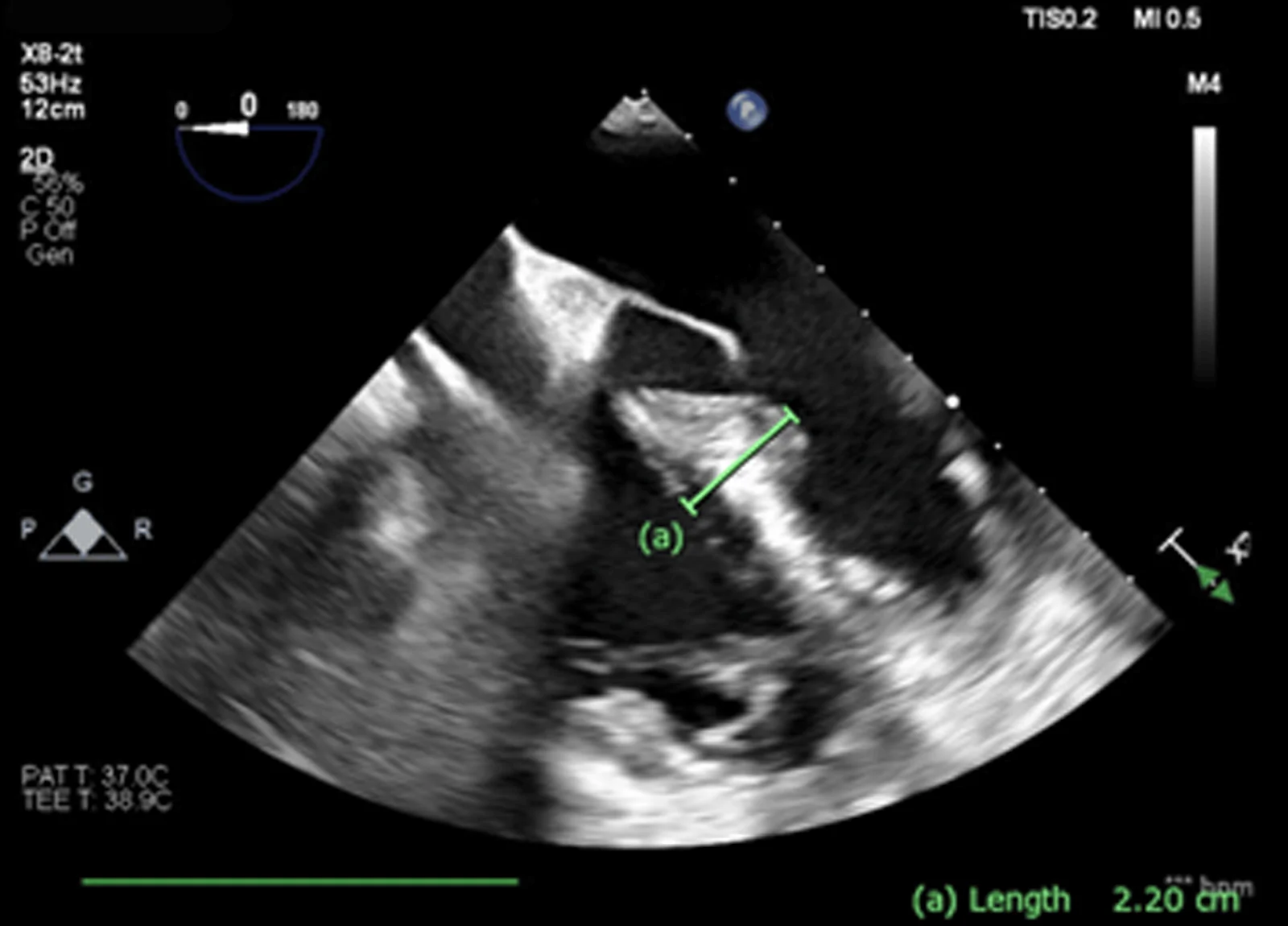
Hypertrophic interventricular septum on intraoperative TEE The mid-esophageal four-chamber view showing a thickness of 2.2 cm (marked as (a)) confirms the interventricular septum hypertrophy TEE: transesophageal echocardiography

The procedure was then performed via a clamshell thoracotomy with sequential bilateral lung transplantation. Due to cardiopulmonary instability during lung explantation and implantation and the patient’s intolerance upon clamping of his pulmonary arteries, partial central venoarterial extracorporeal membrane oxygenation (VA-ECMO) was utilized for intraoperative support as routinely described [7-9].

After bilateral donor lungs were implanted, VA-ECMO was discontinued. The patient initially maintained his vital signs, but about 20 minutes after weaning from VA-ECMO, his blood pressure started to decrease continuously to a mean arterial pressure of around 40-50 mmHg, while his heart rate stayed around 80 beats/minute. There was no increase in peak pressure on the ventilator. He also developed pulmonary hypertension as his mean PA pressure increased from the baseline of 15 to 35 mmHg, which did not respond to inhaled epoprostenol via the endotracheal tube. Typical treatment for hypotension with intravascular fluid, cell saver, norepinephrine, and vasopressin did not stabilize his hemodynamics. Indeed, his profound and refractory hypotension was noted to be exacerbated by boluses of norepinephrine (at typical doses of 8 and 16 mcg), calcium chloride (1 mg), and epinephrine (at typical doses of 10 and 20 mcg), which dropped his mean arterial pressure by 5-10 mmHg consistently with each bolus. Simultaneous TEE imaging revealed a very small left ventricle, SAM of the mitral valve, and moderately reduced right ventricular function with left-sided bowing of the interventricular septum (Figure 2), with no clot appreciated in major pulmonary arteries.

**Figure 2:**
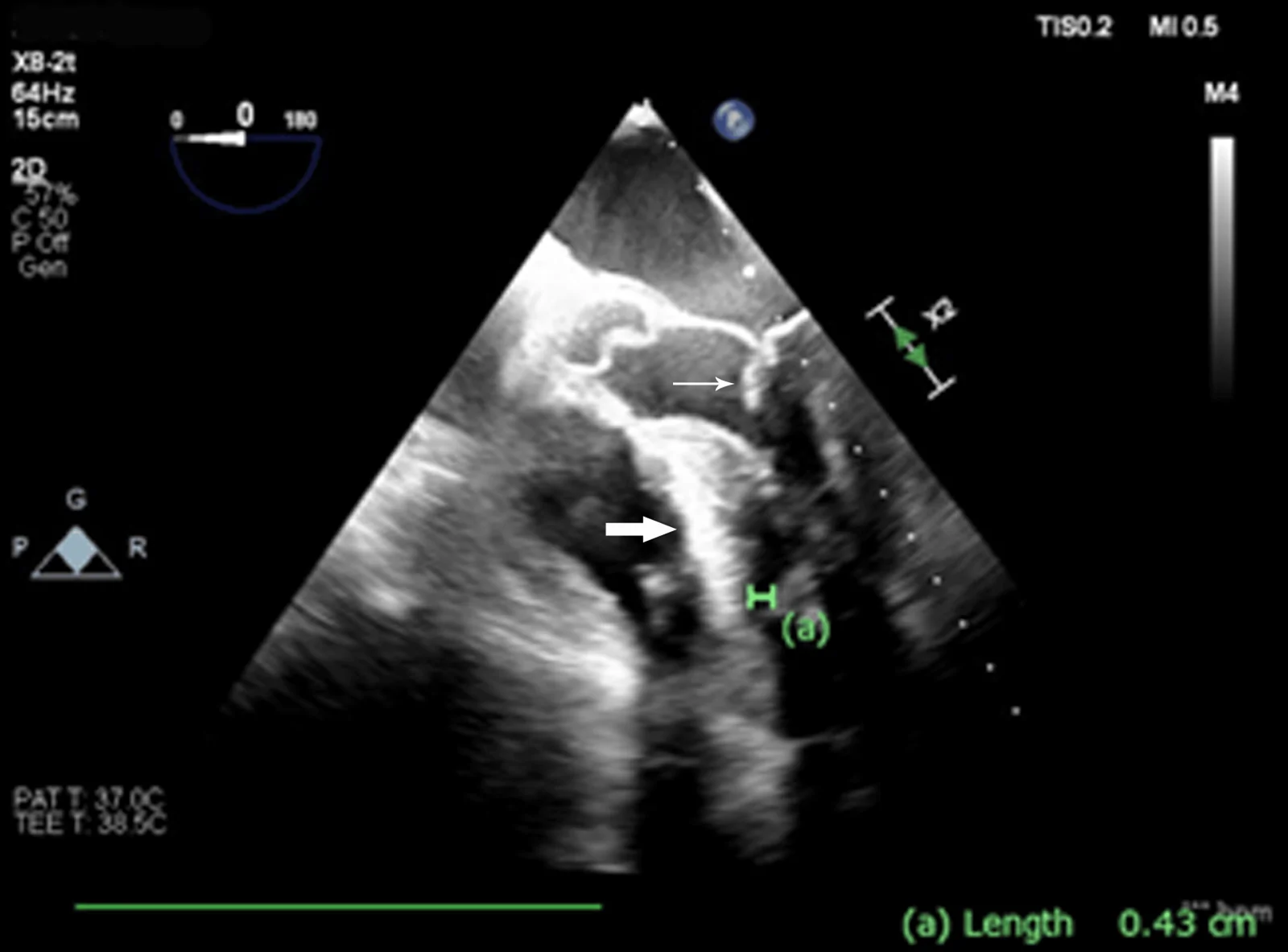
Intraoperative TEE showing SLV SAM of the mitral valve (narrow arrow), acute right heart failure with the interventricular septum bowing to the left side (wide arrow), and a very small left ventricular mid-cavity diameter of 0.43 cm (marked as (a)) are shown in a mid-esophageal four-chamber view TEE: transesophageal echocardiography; SAM: systolic anterior motion; SLV: suicide left ventricle

Realizing that this was most likely an SLV event, 200 mcg of IV phenylephrine was bolused, which increased the patient’s mean arterial pressure by over 10 mmHg within seconds. A phenylephrine drip was then initiated, and the norepinephrine drip was discontinued. The patient’s PA pressure began to decrease with resolution of his right heart dysfunction and SAM on the TEE (Figure 3).

**Figure 3:**
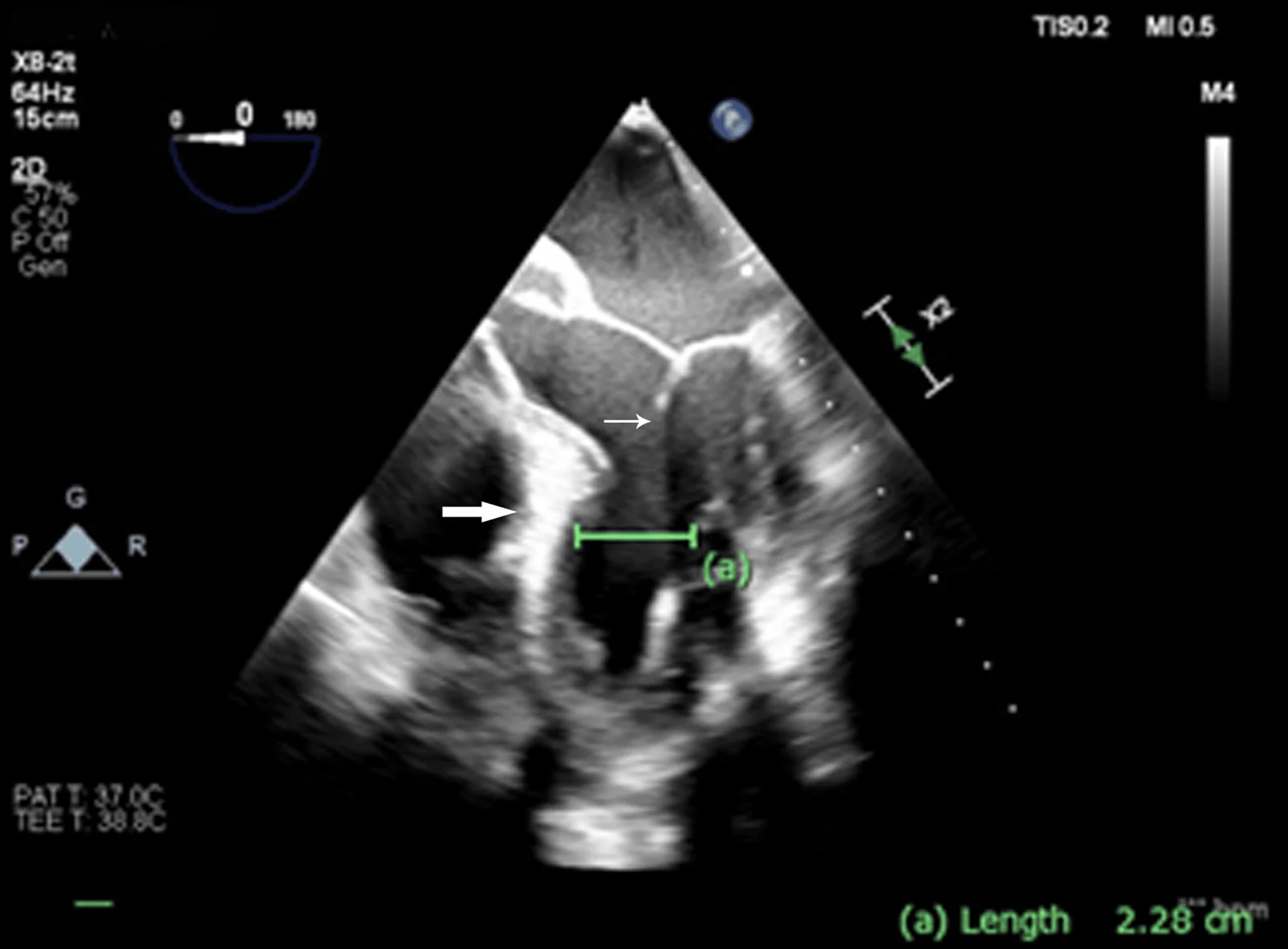
TEE showing resolution of SLV after treatment Resolution of SAM and LVOT obstruction (narrow arrow), neutral position of the interventricular septum (wide arrow), and expansion of the left ventricular mid-cavity diameter to 2.28 cm (marked as (a)) are shown in a mid-esophageal four-chamber view TEE: transesophageal echocardiography; SLV: suicide left ventricle; SAM: systolic anterior motion; LVOT: left ventricular outflow tract

Eventually, his phenylephrine drip was titrated to 200 mcg/minute. This medication alone maintained the patient’s blood pressure within the normal limit. The patient was kept intubated and taken to the intensive care unit on 200 mcg/minute of phenylephrine. He was then successfully extubated on postoperative day 1, and his phenylephrine drip was gradually weaned off within the next 36 hours. This patient had a lengthy postoperative course of 24 days, complicated by newly developed atrial fibrillation, altered mental status caused by cefepime, anemia secondary to chest wall hematoma, and gastroparesis requiring a gastrojejunostomy tube placement, but was successfully discharged home. He has remained on room air, and his follow-ups to date have been normal.

## Discussion

SLV is a rare but potentially life-threatening cause of perioperative hemodynamic collapse resulting from LVOT obstruction following abrupt changes in ventricular afterload. SLV has been mostly reported after transcatheter or surgical aortic valve replacement procedures, where sudden relief of chronic afterload produces a hyperdynamic left ventricle and predisposes to SAM of the mitral valve and LVOT obstruction [10]. Reports of SLV outside of aortic valve interventions are exceedingly limited. To our knowledge, there has only been one case report of SLV post a noncardiac procedure [2]. Our case here is the first report of an intraoperative SLV event in a procedure unrelated to aortic valve manipulation that was diagnosed and treated appropriately to achieve a positive patient outcome.

Development of SLV in our case was most likely multifactorial, driven by both hemodynamic shifts after VA-ECMO weaning and the patient’s underlying myocardial structural characteristics. During VA-ECMO, oxygenated blood is returned to the ascending aorta, ultimately increasing systemic afterload. Upon weaning from VA-ECMO, this retrograde flow is rapidly reduced or eliminated, resulting in a sudden decrease in afterload [11]. This afterload reduction creates a physiologic state analogous to that seen after relief of aortic stenosis, predisposing to hyperdynamic ventricular contraction and LVOT obstruction. In susceptible patients, this transition can precipitate the classic SLV physiology [1,2]. Our patient may also have underlying myocardial features that further increase his susceptibility to LVOT obstruction, as hemochromatosis is associated with myocardial iron deposition that can lead to iron-overload cardiomyopathy, including interventricular septal hypertrophy and diastolic dysfunction [6], both of which were present in our patient. However, because iron-quantification studies are lacking, this remains a hypothetical mechanism. The hypertrophic interventricular septum (Figure 1) revealed on intraoperative TEE is a known risk factor for LVOT obstruction and mitral leaflet displacement [12]. These structural features, combined with a relatively small ventricular cavity and preserved contractility, likely contributed to SLV development when afterload was reduced.

Hypotension during lung transplantation is very common and can have various etiologies such as hypovolemia from hemorrhage or fluid loss, left or right ventricular dysfunction due to strain on the heart, dysrhythmia, systemic inflammatory response syndrome triggered by the surgical procedure or cardiopulmonary bypass, and tension pneumothorax. Diagnosis of SLV can be very challenging, especially in atypical clinical scenarios that do not involve aortic valve intervention, such as after laparoscopic cholecystectomy [2] or during this lung transplant. However, the dire consequences of delayed or missed diagnosis [1,2] require anesthesiologists to keep SLV as an important differential diagnosis for unexpected hemodynamic instability, especially in patients with high-risk factors such as hypertrophy of the interventricular septum and left ventricle or propensity to SAM. TEE monitoring was essential for the timely diagnosis in our case (Figure 2) and also helped with differential diagnosis in the setting of acute right heart failure such as pulmonary embolism (no clot appreciated in major pulmonary arteries), stenosis or kinking of pulmonary vascular anastomosis, tension pneumothorax, or anaphylactic reaction to medications (no change of peak pressure on mechanical ventilation). When TEE is not readily available, TTE and clinical characteristics can also help with diagnosis. For example, paradoxical hypotension in response to inotropic treatment (e.g., epinephrine and calcium) and/or significant response to phenylephrine administration highly suggest the possibility of SLV.

Prompt and appropriate management of SLV is also critical to yield a positive outcome. It focuses on reversing the hemodynamic conditions that exacerbate LVOT obstruction and usually involves increasing preload with volume loading, reducing contractility by discontinuing inotropes and initiating beta-blockers such as an esmolol drip, and augmenting systemic vascular resistance (SVR) [1,2,10]. The first-line vasoconstrictor for SLV is phenylephrine, as it significantly increases SVR without any inotropic effect and simultaneously lowers the heart rate to reduce LVOT obstruction and SAM. A phenylephrine drip was titrated to a very high dose in our case based on clinical effectiveness, which demonstrates its specific role in the treatment of SLV.

In summary, this case report highlights the importance of recognizing SLV in nontraditional settings, particularly during procedures involving rapid hemodynamic shifts such as VA-ECMO use. Timely diagnosis with TEE, TTE, or clinical characteristics, followed by targeted hemodynamic management, is crucial to rapidly reverse the obstruction and prevent progression to severe cardiogenic shock.

## Conclusions

Timely diagnosis and management of SLV can be extremely challenging, especially in atypical situations, yet failure to treat this potentially lethal hemodynamic compromise often leads to dire consequences. Our case report presents an unexpected SLV event and highlights the importance of recognizing risk factors, efficient diagnosing using imaging tools and clinical characteristics, and implementing an effective treatment strategy.

## References

[1] Romano S, D'Andrea E, Cozac DA (2024). Silent threats of the heart: a case series and narrative review on suicide left ventricle post-aortic valve replacement in patients with dynamic LVOT obstruction and aortic stenosis. J Clin Med.

[2] Naseerullah FS, Wickramasinghe SR (2022). Unusual case of post-operative suicide left ventricle in a patient with dynamic LVOT obstruction. J Cardiol Cases.

[3] Lasala JD, Tsai J, Rodriguez-Restrepo A, Atay SM, Sepesi B (2017). Systolic anterior motion of the mitral valve-the mechanism of postural hypotension following left intrapericardial pneumonectomy. J Thorac Dis.

[4] Joshi PK, Patel SC, Shreya D (2021). Hereditary hemochromatosis: a cardiac perspective. Cureus.

[5] Kowdley KV, Brown KE, Ahn J, Sundaram V (2019). ACG clinical guideline: hereditary hemochromatosis. Am J Gastroenterol.

[6] Elmberg M, Hultcrantz R, Simard J, Stål P, Pehrsson K, Askling J (2012). Risk of ischaemic heart disease and cardiomyopathy in patients with haemochromatosis and in their first-degree relatives: a nationwide, population-based study. J Intern Med.

[7] Murray AW, Boisen ML, Fritz A, Renew JR, Martin AK (2021). Anesthetic considerations in lung transplantation: past, present and future. J Thorac Dis.

[8] Elgharably H, Javorski MJ, McCurry KR (2021). Bilateral sequential lung transplantation: technical aspects. J Thorac Dis.

[9] Marczin N, de Waal EE, Hopkins PM (2021). International consensus recommendations for anesthetic and intensive care management of lung transplantation. An EACTAIC, SCA, ISHLT, ESOT, ESTS, and AST approved document. J Heart Lung Transplant.

[10] Chraibi H, Bakamel L, Fellat R, Bendagha N, Moughil S (2023). Conservative management of suicide left ventricle after surgical aortic valve replacement. Cureus.

[11] Luo JC, Zhang YJ, Hou JY, Luo MH, Liu K, Tu GW, Luo Z (2022). Weaning from venous-arterial extracorporeal membrane oxygenation: the hemodynamic and clinical aspects of flow challenge test. Front Med (Lausanne).

[12] Garcia MJ (2016). Constrictive pericarditis versus restrictive cardiomyopathy?. J Am Coll Cardiol.

